# Interaction Between Non-Insulin Glucose-Lowering Medication and Exercise in Type 2 Diabetes Mellitus – New Findings on SGLT2 Inhibitors

**DOI:** 10.3389/fendo.2021.694099

**Published:** 2021-07-15

**Authors:** Christian Brinkmann

**Affiliations:** ^1^ Institute of Cardiovascular Research and Sport Medicine, Department of Preventive and Rehabilitative Sport Medicine, German Sport University Cologne, Cologne, Germany; ^2^ IST University of Applied Sciences, Düsseldorf, Germany

**Keywords:** exercise, training, medication, SGLT2 inhibitor, metformin

## Introduction

Sodium-glucose cotransporter 2 (SGLT2) inhibitors, which block glucose reabsorption in the kidneys, represent a novel class of non-insulin glucose-lowering medication used in the pharmacological therapy of type 2 diabetes mellitus (T2DM). They became available after the introduction of incretin-based therapies with glucagon-like peptide-1 receptor agonists (GLP-1 RAs) or dipeptidyl peptidase-4 (DPP4) inhibitors (1). All these newer drugs have multiple effects that go beyond their glucose-lowering effect (2). It has been concluded in a meta-analysis that patients with T2DM who take SGLT2 inhibitors experience fewer major adverse cardiovascular events and show reduced all-cause mortality (3). Another meta-analysis (including data from patients with T2DM, heart failure or chronic kidney disease) demonstrates that SGLT2 inhibitor use can reduce the risk of cardiac arrhythmia (4). Yet another meta-analysis shows that SGLT2 inhibitor use can reduce blood pressure in T2DM patients (5). The beneficial effects of SGLT2 inhibitors on the cardiovascular system do not only depend on the glucose-lowering effect, but are also based on other possible mechanisms, e.g. increased natriuresis and diuresis [for an overview, see (6)].

Although it is generally accepted that regular exercise also has glucose-lowering and beneficial cardiovascular effects, the combination of non-insulin glucose-lowering medication and regular exercise does not necessarily have to have additive effects on glycemic variables and/or cardiovascular health/cardiorespiratory fitness. By contrast, it has been concluded in some studies involving insulin resistant patients who take metformin, which is often recommended as the first-line medication in T2DM, that certain exercise adaptations (improvements in insulin sensitivity, peak oxygen uptake, blood pressure, lipid profile) are not further increased or can even be attenuated through metformin use (7–11). Details on the underlying molecular mechanisms of how metformin and exercise interact and affect exercise training outcomes have yet to be determined. Among others, changes in (exercise-induced) oxidative stress and/or inflammation (with effects on post-exercise cellular signals) or in (exercise-induced) autophagy have been discussed (10, 12). It should be noted, however, that not all studies report similar or blunted adaptations for all outcomes when exercise training is combined with metformin use. In the study of Viskochil et al. (13), for example, metformin and exercise training, but not exercise alone, lowered proinsulin concentrations and increased insulin clearance in adults with prediabetes. But what about the influence of SGLT2 inhibitors on training outcomes?

## Influence of SGLT2 Inhibitor Use on Exercise Training Outcomes

Newman et al. (14) recently demonstrated in a study involving overweight/obese patients (non-insulin resistant subjects) in a parallel group design (exercise training + SGLT2 inhibition, exercise training + placebo) that SGLT2 inhibitor use can attenuate/block some health-related training outcomes. While for several physiological adaptations/health variables (e.g., body mass index, fat mass, maximal oxygen uptake (VO_2peak_), skeletal muscle citrate synthase activity) a mixed analysis of variance (ANOVA) revealed no time (pre/post-training) * group (SGLT2 inhibition/placebo) interaction, ANOVA showed significant time * group effects for insulin sensitivity (Matsuda index) and fasting blood glucose. Although p values were not reported in post-hoc analyses, changes in mean values indicated a negative influence of SGLT2 inhibitors on these outcome measurements compared with the placebo.

These results contradict the results of a study involving diabetic animals (rodent models for type 2 diabetes mellitus (4 groups: sedentary + SGLT2 inhibition, sedentary + placebo, training + SGLT2 inhibition, training + placebo)) indicating that exercise training + SGLT2 inhibitor administration maximized some positive effects on variables of glycemic control (15). Statistical analyses were performed by two-way ANOVA (to detect main effects and/or possible interactions) and, where appropriate, by post-hoc tests. Fasting glucose, insulin and insulin response (area under the curve) during an oral glucose tolerance test were all significantly lower with exercise training + SGLT2 inhibition than with exercise training alone (significant main effect of drug). The glucose area under the curve was significantly lower in the trained animals taking SGLT2 inhibitors than in the sedentary animals taking SGLT2 inhibitors (significant main effect of exercise). Only SGLT2 inhibition combined with exercise training led to a significantly reduced body weight (compared with the body weight of sedentary animals without SGLT2 inhibition, significant main effect of drug). For other variables (e.g., total cholesterol, triglycerides) there were no differences between groups post-treatment.

## Discussion

The two above-mentioned studies report some contradictory findings on the direction in which SGLT2 inhibitors can affect training outcomes (**Table 1**). A human study involving individuals with type 2 diabetes is still pending. It remains to be seen whether the situation in type 2 diabetes patients is more similar to that of overweight/obese, non-diabetic subjects or to the situation observed in diabetic rodents.

**Table 1:** Contradictory findings on the direction in which sodium-glucose cotransporter 2 (SGLT2) inhibitors can affect training outcomes - Summary table of the data discussed in the present article.

Study	Subjects/Animals	Groups	Relevant outcomes
Newman et al. (14)	Overweight and obese patients with normal glucose control	Training + SGLT2 inhibition Training + placebo	Significant time * group effect for: -insulin sensitivity (Matsuda index) -fasting blood glucose → Beneficial training outcomes were attenuated/blocked through SGLT2 inhibitor use
Linden et al. (15)	Rodent models for type 2 diabetes mellitus	(Sedentary + SGLT2 inhibition) (Sedentary + placebo) Training + SGLT2 inhibition Training + placebo	Significant main effect of drug for: -fasting blood glucose -fasting insulin -insulin response during an oral glucose tolerance test → Beneficial training outcomes were maximized through SGLT2 inhibitor use (compared with training alone)

One reason that may account for a non-additive effect of training and SGLT2 inhibitors could be a higher rating of perceived exertion (RPE) due to decreased carbohydrate availability (14, 16). A higher RPE score could lead to reduced absolute workloads during exercise. Newman et al. (14) found a higher RPE score among patients during early exercise sessions in the SGLT2 inhibitor group compared with the placebo group. No information on absolute workloads is available in their study. However, this is unlikely to be a plausible explanation for the observed outcome differences because the RPE scores during the last training sessions were similar between groups, and the training loads based on an individually calculated training heart rate (and not on the patients’ RPE). Instead, the authors speculatively attribute the increased fasting glucose and absence of improved insulin sensitivity to a possible up-regulation of glucagon in the training + SGLT2 inhibitor group. SGLT2 inhibitors may stimulate the secretion of the insulin counterregulatory hormone glucagon from pancreatic α-cells (mediated by a decrease in the insulin level, but also directly) (17). Other convincing explanations have yet to be found.

On the other hand, it has been demonstrated that SGLT2 inhibitor use can increase mitochondrial oxidative phosphorylation capacity in skeletal muscle (in a murine model of heart failure) (18). Exercise endurance capacity could consequently be increased to a higher extent by physical training. Higher training loads could lead to increased training adaptations.

In connection with the use of SGLT2 inhibitors, it should also be pointed out that the risk of developing (euglycemic) ketoacidosis can be increased (also in connection with exercise) (17, 19). Although not all underlying mechanisms are entirely clear, it can be assumed that different mechanisms play a role. Due to increased urinary glucose excretion, insulin demand and secretion from pancreatic ß-cells are reduced. This can stimulate lipolysis and the production of free fatty acids, which are converted into ketone bodies in the liver. SGLT2 inhibitors may also induce the secretion of glucagon, which can contribute to ketone overproduction through inhibitory effects on acetyl-CoA carboxylase (thereby increasing the rate of ß-oxidation) (17). T2DM patients with ß-cell insufficiency (in old age or with a long history of type 2 diabetes mellitus) or with a longer use of SGLT2 inhibitors may be at higher risk of ketoacidosis (17, 19). In patients being treated with insulin, it could be dangerous when self-administered insulin is reduced by too much or not injected at all. Reasons for why exercise may further increase the risk of ketoacidosis in diabetes patients who take SGLT2 inhibitors could be as follows: during exercise, the glucagon:insulin ratio and secretion of stress hormones (which can also induce a rise in the availability of free fatty acids) can be increased, especially during intense exercise (20). Furthermore, excessive exercise can cause hypohydration/hypovolemia (in case of poor oral fluid intake). This can further drive a rise in stress hormones/insulin counterregulatory hormones and increase ketogenesis (21). Therefore, the administration of SGLT2 inhibitors should always be carefully considered and patients should be informed about possible health risks and how to prevent them. A position statement by the American Association of Clinical Endocrinologists recommends stopping the intake of SGLT2 inhibitors at least 24 hours prior to extreme physical activity (22). For further safety issues related to SGLT2 inhibitors, please see the review of Milder et al. (23).

In general, research on drug-exercise-interaction in diabetes is important for optimizing the therapy of millions of patients who engage in regular physical activity. It would be extremely disappointing if patients exercise for hours on end but do not achieve the desired results because their daily drugs act as a brake. On the other hand, it would be detrimental and dangerous if patients stopped taking their medication because they are participating in an exercise training program and their mortality risk/risk of disease complications increases due to the absence of effects/mechanisms of drugs, which cannot be (sufficiently) induced by regular exercise. Therefore, more observational studies over longer periods of time, also considering all-cause mortality in different cohorts, i.e. training groups with/without non-insulin glucose lowering drugs, are necessary. Furthermore, to be able to give patients sound recommendations, the detailed molecular mechanisms of potential drug-exercise-interactions (in the context of acute exercise as well as regular exercise) must be determined in experimental studies, and questions on the appropriate dose of drugs and exercise, different combinations of various drugs and exercise types or the timing of drug intake and exercise must be addressed first.

## Author Contributions

CB wrote and revised the manuscript.

## Funding

The IST University of Applied Sciences provided funding for the open access publication. CB reports grants and professional fees from Abbott Diabetes Care, outside the submitted work. The funder was not involved in the study design, collection, analysis, interpretation of data, the writing of this article or the decision to submit it for publication.

## Conflict of Interest

The author declares that the research was conducted in the absence of any commercial or financial relationships that could be construed as a potential conflict of interest.
